# A Highly Sensitive NiO Flexible Temperature Sensor Prepared by Low-Temperature Sintering Electrohydrodynamic Direct Writing

**DOI:** 10.3390/mi15091113

**Published:** 2024-08-31

**Authors:** Ting Wang, Xianruo Du, Gaofeng Zheng, Zhiyuan Xue, Junlin Zhang, Huatan Chen, Libo Gao, Wenwang Li, Xiang Wang, Yifang Liu, Jiaxin Jiang

**Affiliations:** 1 School of Mechanical and Automotive Engineering, Xiamen University of Technology, Xiamen 361024, Chinachenht@xmut.edu.cn (H.C.); xmlww@xmut.edu.cn (W.L.); wx@xmut.edu.cn (X.W.); 2 College of Physical Science and Technology, Xiamen University, Xiamen 361102, China; 3 Pen-Tung Sah Institute of Micro-Nano Science and Technology, Xiamen University, Xiamen 361102, China; zheng_gf@xmu.edu.cn (G.Z.); lbgao@xmu.edu.cn (L.G.); 4 Shenzhen Research Institute of Xiamen University, Shenzhen 518000, China

**Keywords:** electrohydrodynamic direct writing, low-temperature sintering, nickel oxide, flexible temperature sensor

## Abstract

Flexible temperature sensors have diverse applications and a great potential in the field of temperature monitoring, including healthcare, smart homes and the automotive industry. However, the current flexible temperature sensor preparation generally suffers from process complexity, which limits its development and application. In this paper, a nickel oxide (NiO) flexible temperature sensor based on a low-temperature sintering technology is introduced. The prepared NiO flexible temperature sensor has a high-resolution temperature measurement performance and good stability, including temperature detection over a wide temperature range of (25 to 70 °C) and a high sensitivity performance (of a maximum TCR of −5.194%°C^−1^ and a thermal constant of 3938 K). The rapid response time of this temperature sensor was measured to be 2 s at 27–50 °C, which ensures the accuracy and reliability of the measurement. The NiO flexible temperature sensor prepared by electrohydrodynamic direct writing has a stable performance and good flexibility in complex environments. The temperature sensor can be used to monitor the temperature status of the equipment and prevent failure or damage caused by overheating.

## 1. Introduction

In smart homes, smart medical and industrial automation, temperature is a basic and essential monitoring parameter. Flexible temperature sensors with their efficient and flexible characteristics have attracted the extensive attention of researchers. The development of a flexible temperature sensor with high sensitivity, a wide temperature range and excellent flexibility is of great significance for the development of intelligent production and human life [1]. The processes used the most for the preparation of flexible temperature sensors are the magnetron sputtering method [2], multi-step lithography [3], pulsed laser deposition of a variety of oxide thin films, such as ZnO, TiO_2_, Al_2_O_3_, etc. [4,5] or inductively coupled plasma etching [6]. Various flexible temperature sensors have been successfully prepared by these methods with excellent temperature sensing capability and mechanical robustness. However, the complexity and high fabrication cost have greatly hindered the practical application of flexible temperature sensors [7,8]. Therefore, there is an urgent demand for a process with a method of preparing thin sensitive films at a relatively low cost and through an easy process.

Jaeho Shin et al. [9] achieved a seamless monolithic structure by simultaneously integrating metal electrodes and metal oxide sensing channels through a single material, using a novel monolithic laser-induced reduction sintering scheme. However, this process usually requires annealing at temperatures above 600 °C, which is incompatible with most flexible substrates. Jin Chai et al. [10] prepared flexible temperature sensors with a stable performance, using a screen-printing process based on carbon nanotube/graphene conductive paste. It has a response time of 2.95 s. The temperature coefficient of resistance was −0.17% °C^−1^ in the range of 12.5–93.7 °C, which was not affected by bending deformation or humidity change. However, the sensitivity and stability of the temperature sensors prepared by this method need to be further improved. Lu Y et al. [11] mixed sensing nanomaterials with liquid polymer substrate precursors to obtain composite elastomers. However, the proportion, particle size and dispersion of the nanomaterials can significantly affect the conductivity and sensing properties of the composite elastomers, thus becoming an unstable factor for the device fabrication and application. 

Nickel oxide (NiO) is an important transition metal oxide with a high temperature coefficient of resistance (TCR) that changes significantly with temperature, providing an excellent temperature detection performance. This makes it an ideal thermally sensitive semiconductor material [12]. With these appropriate properties, flexible temperature sensors with high sensitivity and a wide temperature range can be fabricated. Researchers have explored the use of NiO for flexible temperature sensors despite posing some challenges. For example, In the effect of calcination temperature on the performance of NiO sensors, it was noted that the response time and recovery time at an operating temperature of 250 °C were 56 s and 21 s, respectively. This study may be important for improving the response time of temperature sensors based on nickel materials [13]. NiO flexible temperature sensors obtained by monolithic laser-induced reduction sintering methods have been reported to monitor heat signals from the human body, the sensitivity showing potential for e-skin applications [14]. However, the defects of PVP materials, such as poor stability and low mechanical strength, as well as large operating resistance and high post-processing temperatures, severely limit the performance and reliability of such sensors in practical use [15]. Therefore, in order to improve the practicality of NiO flexible temperature sensors, it may be necessary to explore or develop new manufacturing technologies and materials to address these issues.

As an important method for one-dimensional material preparation, electrohydrodynamic direct writing has the advantages of simple operation, low cost and controllable morphology, thus enabling the preparation of fiber-structured materials [16,17]. The electrohydrodynamic direct-writing technology is capable of fabricating complex one-dimensional ultra-long structures with micrometer or even nanometer resolutions with a high specific surface area and porosity [18]. By using the electrohydrodynamic direct-writing technology, Kyung Hyun Choi et al. [19] used 55 wt% of silver nanoparticle ink operated on a roll-to-roll (R2R) system to optimize parameters, such as print head speed to form a continuous line of micro-nano-metallic structures. The temperature sensor obtained by this technique has a temperature coefficient of resistance (TCR) of 0.0768% °C^−1^. By optimizing material formulations and sintering conditions, high conductivity structures can be achieved, which is essential for manufacturing high-performance electronic devices. In our previous works, low-temperature sintering electrohydrodynamic direct-writing technology is proposed as an advanced manufacturing process that combines the advantages of low-temperature sintering and electrohydrodynamic direct writing. The low-temperature sintering process avoids thermal damage to sensitive materials, making it particularly suitable for the processing of flexible electronics and biodegradable materials [20,21]. At the same time, the diameter, arrangement and the stacking of fibers can be customized as needed, thereby adjusting the electrical and mechanical properties of the devices [22].

In this paper, the low-temperature sintering electrohydrodynamic direct-writing method is used to prepare a NiO flexible temperature sensor with a unique fiber structure. A 50 °C low temperature sintering post-processing can be obtained with a smooth surface morphology of the fiber at the same time to preserve a certain amount of polyethylene oxide (PEO), enhance the adhesion of the metal structure and flexible substrate and ensure the stability of the electrical properties of the flexible sensor. The sensor not only has the advantages of high resolution, a wide temperature range and good flexibility, but also has good stability, which is of great significance for smart homes and smart medical and industrial automation.

## 2. Materials and Methods

### 2.1. Experimental Materials

The materials used in the experiment were nickel acetate tetrahydrate [Ni(CH_3_COO)_2_·4H_2_O, with a purity of 99% and a molecular weight of 249.08], isopropanolamine (CH_3_CH(OH)CH_2_NH_2_, with a purity of 99% and a molecular weight of 75, from Shandong Yousuo Chemical Technology Co., Ltd., Linyi, China), anhydrous ethanol (C_2_H_5_OH, AR, with a molecular weight of 46.07, from China National Pharmaceutical Group Chemical Reagent Co., Ltd., Beijing, China), polyethylene oxide (PEO, average molecular weight = 300,000 g/mol, Dadi Fine Chemical Co., Ltd., Tianjin, China) and deionized water.

The one-pot synthesis is a chemical reaction strategy that improves reaction efficiency by allowing multiple reaction steps to occur in a single reactor. In this experiment, 0.25 g of Ni(CH_3_COO)_2_·4H_2_O and 0.65 g of PEO were dissolved in 1.4 g of deionized water, 2.3 g of ethanol and 0.4 g of CH_3_CH(OH)CH_2_NH_2_. Then, the mixed solution was stirred for 24 h in a thermostatic magnetic stirrer to obtain the precursor solution.

### 2.2. Experimental Apparatus

A hollow dispensing needle with an inner diameter of 0.11 mm was used as a nozzle. The positive output of a DC high-voltage power supply (DW-P503-1ACH1, Tianjin Dongwen High-voltage Power Supply Co., Ltd., Tianjin, China) was connected to the nozzle, and the negative output was connected to a grounded collection plate to form a spatial high-voltage electric field between the nozzle and the collection plate. A precision syringe pump (Pump 11 Pico Plus Elite, Harvard, USA) continuously supplied the solution to the nozzle at a constant rate. The collection plate was fixed on a precision 2D motion platform (REI 95LM-050, Suzhou Gaohe Equipment Control Technology Co., Ltd., Suzhou, China), and its trajectory and speed were controlled by a host computer. A CCD camera (uEye UI-2250SE-C-HQ, IDS, Berlin, Germany) was used to observe and record the solution rheology and jet ejection process. A field emission scanning electron microscope (SUPRA55 SAPPHIRE, Zeiss Optical Instruments, Oberkochen, Germany) was used to observe the microstructure morphology. An electronic analytical balance (Sartorius Scientific Instruments Beijing Co., Ltd., Beijing, China) and a constant temperature magnetic stirrer (C-MAG HS7, IKA, Staufen, Germany) were used to prepare the solution. The sintering of materials and the performance testing of the sensor were conducted using a constant heating table (DB-XAB, Shanghai Yixin Scientific Instrument Co., Ltd., Shanghai, China).

### 2.3. Preparation of NiO Flexible Temperature Sensor

**Figure 1:**
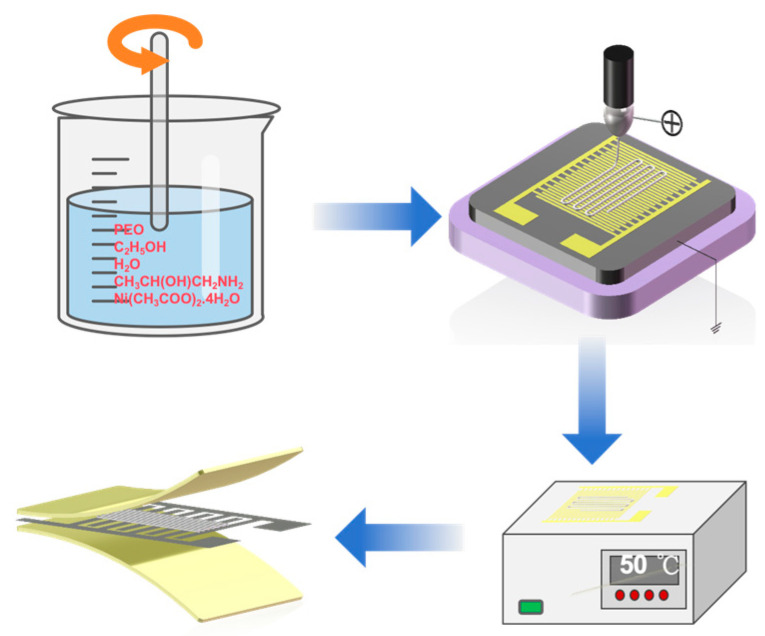
Preparation process of NiO flexible temperature sensor.

The prepared precursor solution was transferred into a 1 mL plastic syringe, and then the syringe was fixed to the syringe pump with a feeding rate of 1 μL/h. The stainless-steel needle was connected to the positive electrode of the high-voltage power supply, and the receiver plate was connected to the negative electrode. The distance between the nozzle and the receiver plate was 1 mm, and the applied voltage was 1.46 kV. An interdigital electrode served as the negative receiving plate to collect the direct-writing structure, of which the size of the sensitive area was 5 mm × 10 mm. The electrode width was 50 μm, the electrode spacing was 50 μm, and there were 15 pairs of fork fingers on each interdigital electrode. As the polymer solution was pushed out of the nozzle, it tended to form spherical droplets with the action of surface tension. When the high voltage was applied to the surface of the droplets, the electrostatic repulsion was strong enough to cause the droplets to form a conical shape, i.e., a Taylor’s cone. When the electric field strength was sufficiently large, a jet was formed at the tip of the Taylor cone. Subsequently, the jet was stretched and thinned and was ultimately deposited onto the collecting plate as nanofibers. The fiber collected from the negative plate was heated with a heating table at 50 °C for 1 h to obtain a NiO fiber. A Polyimide (PI) film with a thickness of 0.05 mm was used as the encapsulation material, and the PI film was cut into a rectangle at the size of 10 mm × 20 mm. Copper wires were used as conductors to connect to the ends of the fork finger electrodes with nickel oxide film to complete the preparation of a 10 mm × 20 mm flexible temperature sensor with a thickness of 0.11 mm. Since the PI is stable over a wide temperature range of (−269 °C to 400 °C) and has high mechanical strength and flexibility, its excellent water resistance and hydrophobicity help maintain performance and reliability in humid environments. In addition, the assembled temperature sensors are easy to deform without causing a structural collapse, giving the sensing element excellent flexibility. The preparation process of the NiO flexible temperature sensor is shown in Figure 1. 

## 3. Results and Discussion

### 3.1. Fiber Characterization

**Figure 2:**
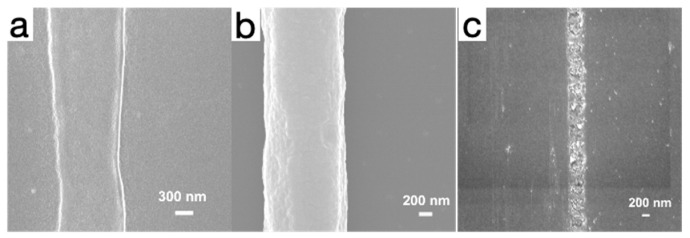
(**a**) SEM image of fiber without sintering. (**b**) SEM image of fiber after sintering at 50 °C. (**c**) SEM image of fiber after sintering at 100 °C.

The synthesis process of the material and the morphological characteristics of the final fiber structure is investigated. Scanning electron microscopy (SEM) is used for characterization. Scanning electron microscopy (SEM) is a high-resolution imaging technique that provides microscopic details of the surface morphology of materials. Figure 2a shows the SEM image of the unsintered treated fiber. From the image, it can be seen that the surface of the fiber is rough. Figure 2b shows the SEM images of the fibers after sintering at a temperature of 50 °C. The surface of the fibers after sintering at 50 °C becomes significantly smoother compared to the unsintered fibers, indicating that sintering at a lower temperature helps in the repair of defects on the surface of the fibers and the densification of the structure due to sintering at a lower temperature. This smooth surface morphology contributes to the chemical stability of the fibers. The fiber diameter obtained after sintering at 50 °C was 1.340 μm. Due to the smaller diameter of the fibers, a finer temperature measurement resolution can be provided, enabling accurate monitoring of temperature changes. At the same time, the very fine feature size of fibers prepared by the low-temperature sintering electrohydrodynamic direct-writing technology makes it highly promising for the manufacture of high-precision electronic components. Figure 2c shows the SEM image of the fibers after sintering at a higher temperature of 100 °C. Compared with the fibers sintered at 50 °C, the liquid phase substances such as water and anhydrous ethanol gradually evaporate as the temperature increases, resulting in a discontinuous agglomerate morphology of the fibers. When the temperature exceeds 60 °C (above the melting point of PEO), PEO enters an amorphous state, resulting in the conductive fibers losing their fibrous structure and melting, and the fibers are dispersed at a sintering temperature of 100 °C. By observing the morphology of fibers at different sintering temperatures, it is evident that the sintering temperature significantly affects the fiber structure. This impact is not only reflected in the surface morphology of the fibers but also extends to their physical and chemical properties, thereby significantly influencing the application performance of the material. Completing the sintering process at lower temperatures helps protect heat-sensitive flexible substrates and other materials, an advantage that is particularly crucial for application in flexible electronic devices and plastic materials. Through this low-temperature sintering technology, a sophisticated and adaptable electronic component is expected to be achieved, broadening the scope of material applications.

**Figure 3:**
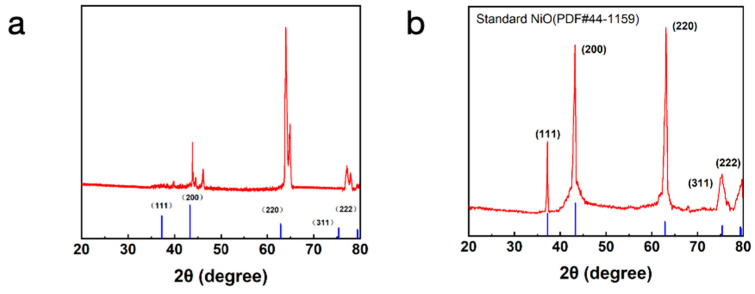
(**a**) XRD spectra of fiber without low-temperature sintering. (**b**) XRD spectra of fiber sintered at low temperatures.

X-ray diffraction (XRD) is an important characterization technique used to analyze the crystal structure and phase composition of materials. Figure 3a demonstrates the XRD spectra of fibers that have not been cryo-sintered, while Figure 3b shows the XRD spectra of fibers that have been cryo-sintered at 50 °C. A comparison of Figure 3a,b shows that nickel oxide (NiO) is produced after low temperature sintering.

In Figure 3b, the XRD spectra after low-temperature sintering show typical diffraction peaks of nickel oxide (NiO). These peaks correspond to the (111), (200), (220), (311) and (222) crystal planes of NiO, respectively. The sharpness and intensity of these diffraction peaks indicate that the low-temperature sintering process successfully promoted the formation of the nickel oxide phase. 

Nickel oxide is a material with a high electronic conductivity and catalytic activity and is widely used in the field of sensors. The advantage of low-temperature sintering technology is its ability to promote material densification and phase formation at lower temperatures while reducing energy consumption and production costs. In addition, low-temperature sintering helps to maintain the microstructure of the material, avoiding grain growth and destruction of the material structure that may occur during high-temperature sintering.

### 3.2. Performance of NiO Flexible Temperature Sensor

**Figure 4:**
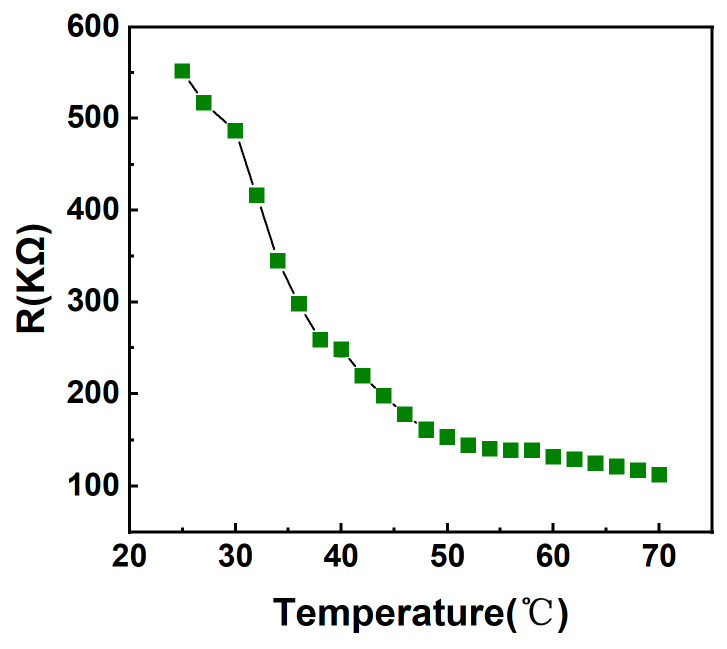
Variation curve of resistance with temperature.

The sensor has a very small change in intrinsic resistance and stable electrical properties in the bending state. In order to analyze the response of the senor to temperature changes, the temperature sensor was fixed to a temperature control platform, and a conductive tape was used to ensure close contact between the sensor and the platform. By varying the applied voltage, it is possible to regulate the temperature on the temperature-controlled platform, resulting in a continuous temperature change from 25 °C to 70 °C. During this process, the resistance value of the sensor exhibits the characteristic of decreasing with increasing temperature, specifically from 552.1 kΩ to 112.1 kΩ. The flexible temperature sensor exhibits typical negative temperature coefficient (NTC) semiconductor characteristics as shown in Figure 4.

**Figure 5:**
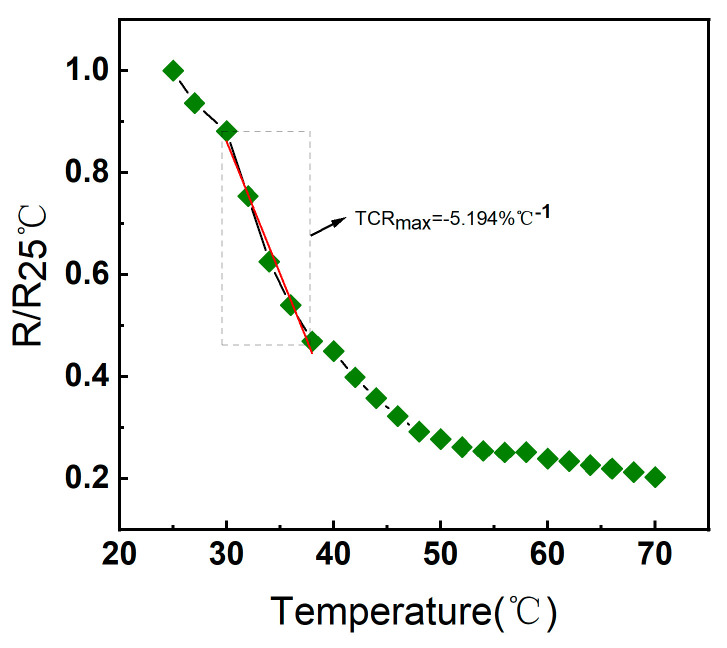
Relative resistance variation of NiO flexible temperature sensor at a temperature of 25–70 °C and the corresponding fitted TCR values.

In addition, the temperature coefficient of resistance (TCR), which reflects the temperature sensitivity of the device, can be explained by the following expression [23,24]:

(1) $$\;TCR=\frac{R_{T}-R_{0}}{R_{0}}\cdot \frac{1}{\Delta T}\times 100\%$$

where *R_T_* and *R*_0_ are the resistance measured through the sensor at the applied and ambient temperatures, respectively, and Δ*T* is the temperature change. Negative Temperature Coefficient (NTC) sensors exhibit different Temperature Coefficient of Resistance (TCR) at different temperature stages, which is primarily due to the characteristic changes in the resistivity of the material with temperature variations. By fitting the normalized resistance with respect to the room temperature (R/R25 °C), as shown in Figure 5, at the temperature range of 25 °C to 70 °C, the maximum value of the Temperature Coefficient of Resistance (TCR) is −5.194% °C^−1^ (30–38 °C). From this, it is understood that temperature sensors manufactured using low-temperature sintering electrohydrodynamic direct-writing technology exhibit excellent sensitivity characteristics due to their unique manufacturing process and material selection.

For semiconductor materials, the resistance is nonlinear with increasing thermistor temperature, which can be explained by the following expression [25,26]:

(2) $$R=R_{0}\exp\left(\;\frac{E_{a}}{K_{b}\cdot T}\;\right)=R_{0}\exp\left(\frac{B}{T}\right)$$

where *B* is the factor of thermal sensitivity, *R* is the resistance, *E_a_* reflects the energy demand of a material under specific conditions, *R*_0_ represents the resistance value of the thermistor at the reference temperature, *K_b_* is the Boltzmann constant, and *T* is the absolute temperature. The *B* value defines the relationship between the thermistor’s resistance and temperature, which represents the material constant over a specific temperature interval. The expression in Equation (2) is modified as:

(3) $$InR=In\left(R_{0}\right)+\frac{B}{T}$$

**Figure 6:**
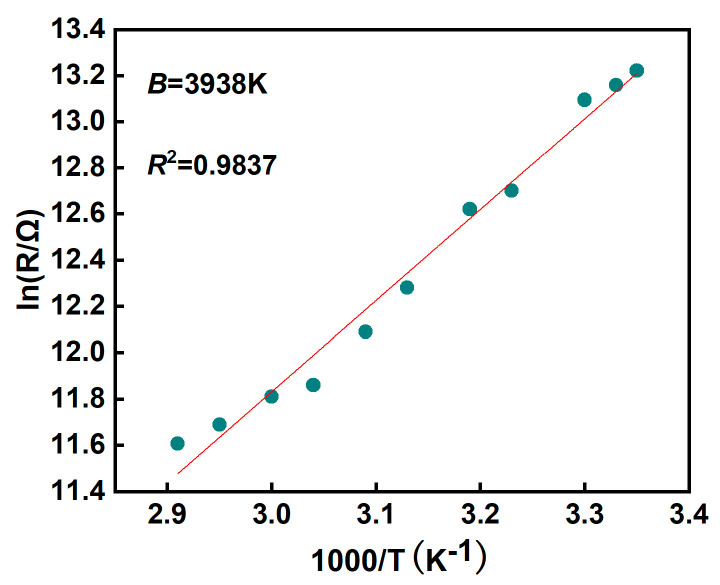
Fitting results of thermistor constants.

Figure 6 illustrates the relationship between the natural logarithm of the resistance (ln*R*) of the temperature sensor and the reciprocal of the absolute temperature (1000/*T*). As shown by the red line in the figure, the fit goodness is close to 0.9837. The results indicate that ln*R* is linearly related to 1000/*T*. This linear behavior is important in practical applications because it simplifies the post-measurement processing procedure.

**Figure 7:**
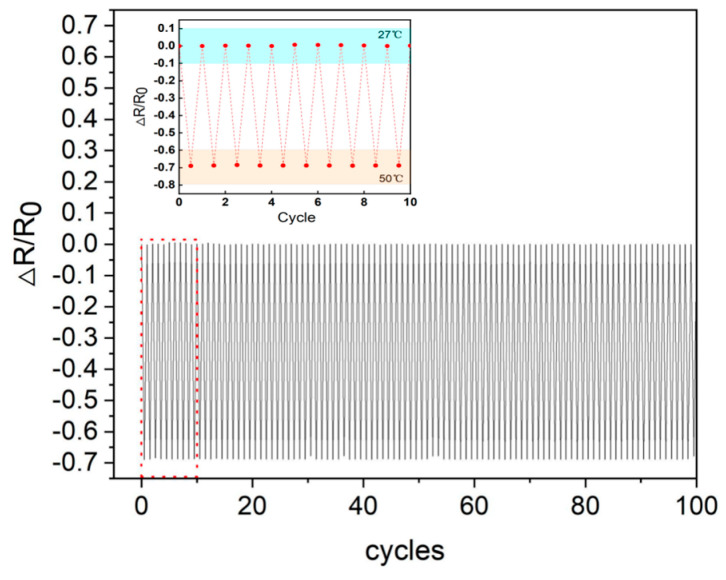
Temperature response of NiO flexible temperature sensor for repeated cooling and heating cycles from 27 to 50 °C.

To assess the reliability and stability of the temperature sensor, it was subjected to 100 cycles of heating and cooling to evaluate its endurance under continuous thermal stress. Among these tests, the first 10 cycles were selected, and the temperature changes during these cycles were displayed in detail and magnified to more clearly observe their performance (Figure 7). The sensor showed good stability. This type of testing is critical to assessing the stability and reliability of the sensor under extreme temperature changes. By simulating the environmental conditions likely to be encountered in real-world applications, it is possible to ensure that the sensor maintains its performance over a long period of operation. During testing, the sensor demonstrated an excellent full-time response. It means that regardless of temperature changes, the sensor is able to provide temperature readings without performance degradation or delay. The rapid and consistent responsiveness is critical for systems that require real-time temperature monitoring, such as in industrial automation, environmental monitoring or medical devices.

In addition, after 100 tests, the sensor showed no signs of performance degradation. It demonstrates high quality of design and manufacturing, capable of withstanding extreme changes in temperature without loss of accuracy or reliability. This durability enables the sensor suitable not only for laboratory environments but also for more demanding field conditions.

**Figure 8:**
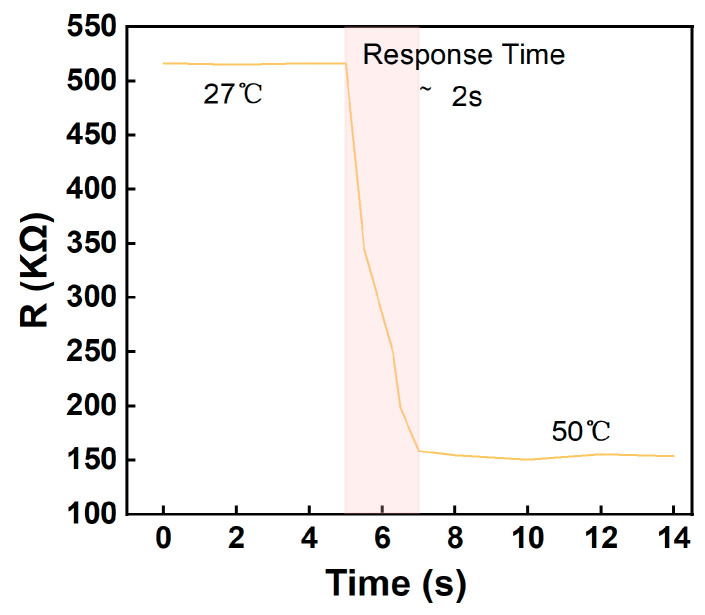
Response time from 27 °C to 50 °C of NiO flexible temperature sensor.

The NiO flexible temperature sensor is able to quickly transfer heat to the sensing element due to its good thermal conductivity, resulting in a fast response to temperature changes. The compact design of the sensor also contributes to reducing the length of the heat transfer path, resulting in a faster response time. As shown in Figure 8, the response time from 27 °C to 50 °C is only 2 s. The fine fibers produced by low-temperature sintering and electrospinning have a faster thermal response time, enabling a rapid detection of temperature changes. This helps to improve the response speed of temperature sensors to temperature fluctuations, which is an advantage over traditional thermometers. The response time of traditional thermometers is affected by a number of factors, including the type, size and material of the temperature sensor, as well as the type of the medium and flow rate. For example, with air-packed mechanical thermometers, the response time for a rise from 10 °C to 90 °C typically takes about 30 s. It shows that the sensor can be used in monitoring human physiological information, industrial process control, automotive engine management systems and medical devices.

**Figure 9:**
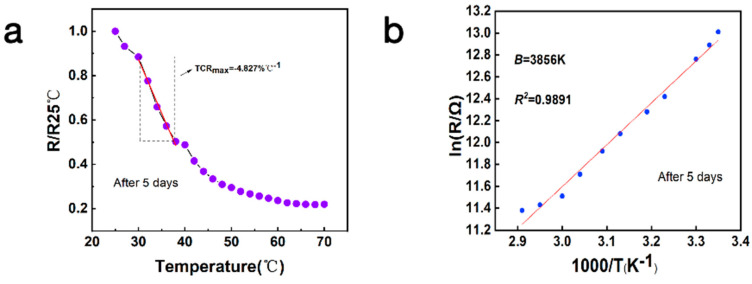
(**a**) Relative resistance changes of the device and the corresponding fitted TCR values after five days at temperatures from 25–70 °C. (**b**) Corresponding fitted results for the thermistor *B* constant.

The TCR_max_ and *B* values of the sensor remain essentially stable with minimal change after being placed under ambient conditions for five days. As shown in Figure 9a, within the temperature range of 25 °C to 70 °C, the maximum values of TCR are −4.827% °C^−1^, respectively. As depicted in Figure 9b, the fitted value for *B* is 3856 K. The test results confirm that the NiO flexible temperature sensor has good chemical stability. Proper encapsulation and the rationality of the device structure ensure the long-term stability of the sensor under the influence of environmental factors. The sensitivity index of the temperature sensor shows good continuity, with no significant changes observed during the environmental test. Devices fabricated by low-temperature sintering electrohydrodynamic direct writing can operate stably under various environmental conditions, including stability in the face of humidity and temperature changes.

To better understand the strengths and limitations of existing technologies, this study conducted a comprehensive evaluation of several different types of temperature sensors as shown in Table 1 [27,28]. Based on the results of the comparative analysis, it was known that although existing sensors performed well in some aspect, they often needed to trade off sensitivity, a working temperature range and cost-effectiveness. This trade-off situation not only limited the maximization of sensor performance, but also restricted their application potential in a broader range of scenarios. On the contrary, the NiO temperature sensor developed in this study demonstrates a comparable Temperature Coefficient of Resistance (TCR) and a larger B value across a broad operating temperature range. In addition, the electrohydrodynamic direct-writing technology does not require expensive equipment and complex process flows. Low-temperature sintering reduces the dependence on high-temperature equipment, reducing manufacturing costs and improving material utilization rates.

**Figure 10:**
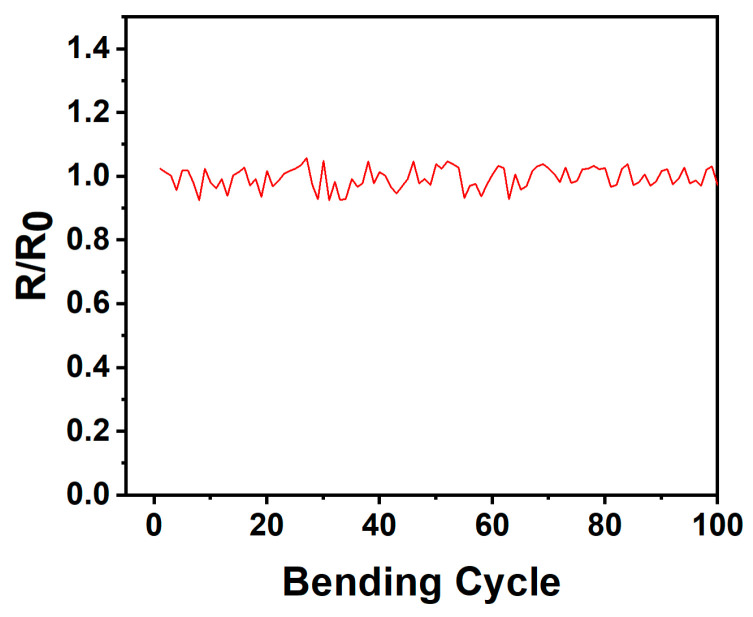
100-cycle repeated tensile bending.

In order to check the mechanical flexibility of the sensor, the NiO temperature sensor is subjected to 100 mechanical performance tests at the same bending angle (45°) and the same temperature (25 °C) as shown in Figure 10. It is clear from the figure that the NiO temperature sensor maintains almost the same resistance under repeated cycles of bending. This indicates that the sensor not only demonstrates excellent stability under mechanical deformation but also possesses outstanding flexibility, enabling it to adapt to the curves and movements of the human body, making it highly suitable for applications in wearable devices and flexible electronic products. The small changes seen in the temperature sensor during the bending test may be the result of experiencing mechanical stresses that result in small changes in the internal structure of the sensor, and these small structural changes may affect the resistive characteristics of the sensor, resulting in small changes in resistance.

## 4. Conclusions

In summary, to address the complexity in the manufacturing process of flexible temperature sensors, this research has successfully developed NiO flexible temperature sensors using the electrohydrodynamic direct-writing technology. The preparation process of 50 °C low temperature sintering, effectively ensures that the NiO fiber morphology is intact, while avoiding high temperatures caused by the detachment of the sensitive material, to provide a guarantee for the stability of the performance of the sensor. The use of PI film as the encapsulation material enhances the thermal conductivity of the sensor. The prepared NiO flexible temperature sensor exhibits high sensitivity (a maximum TCR of −5.194% °C^−1^, *B*-value of 3938 K), wide temperature range characteristics (25–70 °C) and a fast response time (the response time from 27 °C to 50 °C is only 2 s). The device demonstrated excellent repeatability and reliability after 100 heating and cooling cycles. The sensor also showed a small change in resistance under 100 cycles of bending. The miniaturization and flexibility of the sensor makes it easy to integrate with existing smart devices and systems, supporting the development of micromachines. The flexible temperature sensor can be used in smart systems such as smart homes, smart healthcare and industrial automation for smarter and more automated temperature control and management. It can also be used for safety and environmental monitoring in safety monitoring areas such as battery management systems and fire warning systems. The application of the sensor will help to improve the safety and reliability of the system. Future research will further optimize the performance of the sensors and multifunctional integration to develop multifunctional flexible sensors capable of simultaneously detecting multiple physical or chemical parameters, such as integrated temperature, pressure or gas sensors, to achieve more comprehensive monitoring capabilities.

## Figures and Tables

**Table 1:** Comparison of different temperature sensors.

Materials	Process	B-Index (K)	Sensitivity (%/°C)	Temperature Range (°C)	Ref.
SiC	Screen printing	502	−0.556	25–170	[29]
Carbon nanotubes	Screen printing	–	−0.4	−40–100	[30]
f-rGO	Inkjet printing	–	−1.64	25–83	[31]
Graphene	CVD	100	−0.12	−20–100	[32]
Polyvinyl chloride/carbon black	Screen printing	–	−0.148	18–44	[33]
Ag & PEDOT: PSS	Inkjet printing	–	−1.39	25–45	[34]
MWCNTs	Inkjet printing	1135	−1.04	25–50	[35]
BiFeO_3_ + 3.5 wt% graphene	Screen printing	–	−0.961	25–170	[22]
ZrAlN	Sputtering	3000	–	25–100	[36]
NiO/CBNPs	3D Printing	–	−0.111	20–70	[37]
NiO	Electrohydrodynamic direct writing	3938	−5.194	25–70	This work

## Data Availability

The data that support the findings of this study are available upon request from the corresponding author upon reasonable request.
